# The Invasion of the Dwarf Honeybee, *Apis florea*, along the River Nile in Sudan

**DOI:** 10.3390/insects10110405

**Published:** 2019-11-15

**Authors:** Mogbel A. A. El-Niweiri, Robin F. A. Moritz, H. Michael G. Lattorff

**Affiliations:** 1 Department of Biology, King Khalid University, Abha, Asir Region 61321, Saudi Arabia; mogbel7@hotmail.com; 2 Institut für Biologie, Molekulare Ökologie, Martin-Luther-Universität Halle-Wittenberg, 06099 Halle (Saale), Germany; robin.moritz@zoologie.uni-halle.de; 3 Department of Bee Research, National Centre for Research (NCR), Khartoum 11111, Sudan; 4 German Centre for Integrative Biodiversity Research (iDiv) Halle-Jena-Leipzig, Deutscher Platz 5e, 04103 Leipzig, Germany; 5 International Centre of Insect Physiology and Ecology (ICIPE), Nairobi PO Box 30772-00100, Kenya

**Keywords:** *Apis mellifera*, microsatellite DNA, competition intensity, mating frequency, population density

## Abstract

The spread of the dwarf honeybee, *Apis florea*, in Sudan along the river Nile in a linear fashion provides a good model for studying the population dynamics and genetic effects of an invasion by a honeybee species. We use microsatellite DNA analyses to assess the population structure of both invasive *A. florea* and native *Apis mellifera* along the river Nile. The invasive *A. florea* had significantly higher population densities than the wild, native *A. mellifera*. Nevertheless, we found no indication of competitive displacement, suggesting that although *A. florea* had a high invasive potential, it coexisted with the native *A. mellifera* along the river Nile. The genetic data indicated that the invasion of *A. florea* was established by a single colony.

## 1. Introduction

Non-native species that spread in their new, non-native range are invasive species. Often, these species are introduced into their new distribution range by human activities, and sometimes their spread within the new environment is also facilitated by human interventions. Invasive species are characterized by a set of traits that promote their invasive success, e.g., high reproductive rate and a generalist lifestyle [1]. The consequence of biological invasions can be detrimental when native species are affected due to competition or the spill over of diseases [1].

The success of an invasion is determined by different factors. The number of introductions is a predictor for the success [2], but the genetic constitution of the invading species is also of high importance [3]. Introduced species are exposed to different selective forces than in their native range, so the genetic variation for responding to the selective forces needs to be present. This is often difficult to achieve, as introduced populations are small and the introduction into the new range represents a genetic bottleneck for the population [4]. This effect is also known as the paradox of invasive species, namely describing their success despite a low genetic diversity [5,6,7].

Social insects comprise less than 2% of all described insect species [8], but according to the Global Invasive Species Database [9], out of the total 81 invasive insect species, 26 (=32%) are social insects. Invasions of social insects into new and non-native ranges can have detrimental effects. The red imported fire ant (*Solenopsis invicta*), originating in South America and introduced into the south of the USA in the 1930s [10], as well as the introduction of the Argentine ant (*Linepithema humile*) into southern Europe in 1895, resulted in changes in the social structure of the species, which facilitated their spread in the new environment [11]. These examples show the potential for the rapid adaptation of social insects within their new environment.

The genus *Apis* comprises of different species including the cavity-nesting *Apis mellifera*, *A. cerana*, *A. nigrocincta*, and *A. koschevnikovi*; the open nesting dwarf honeybees *A. andreniformis* and *A. florea*; and the giant honeybees *A. dorsata* and *A. laboriosa*. The Western honeybee, *A. mellifera*, is endemic to Europe, Africa, and western Asia [12,13], while all other species are endemic to Asia [13,14], where they often occur in sympatry.

Global invasions of bee species have been studied in detail for the Western honeybee, *A. mellifera*, which has been introduced by humans into several regions, either on purpose (European honeybees into North and South America) or accidentally (African honeybees into South America) [15]. Another invasive species is *A. cerana*, the Eastern honeybee. This species, native to southern Asia, has spread to Papua-New Guinea, Australia, and Solomon Islands [16].

The introduction of honeybees into new continents can have both beneficial and harmful consequences depending on the regional ecological context of the introduction. For instance, the introduction of the European *A. mellifera* to America by European settlers is believed to have been harmless to the local ecosystems [15]. The establishment of apiculture with European honeybees in the new world outweighed any potential negative impact [15]. In contrast, the introduction of African *Apis mellifera scutellata* bees into Brazil in the 1950s caused the Africanized bee problem, with highly aggressive honeybees spreading up to the southern parts of the United States within a few decades [17]. Although honey production increased, there were severe negative effects on the public due to repeated uncontrolled stinging events [18]. In contrast to the negative public perception, the ecological impact of the Africanized honeybee surprisingly remained remarkably small. Even meticulously detailed studies failed to detect major effects on the abundance of native wild bees and insect fauna after the arrival of the Africanized bees [19]. Recent studies show that overall pollination webs are stabilized by introduced *A. mellifera* [20].

The introduction of *A. mellifera* into Asia caused more severe problems resulting from the interactions with other honeybee species. Apart from local resource competition [21], factors such as mis-mating between *A. cerana* drones and *A. mellifera* queens occurred, resulting in sterile queens [22], reviewed in [15]. Particularly disadvantageous was the exchange of pests, parasites, and pathogens between native and introduced honeybee species. Most ill-famed is the parasitic mite *Varroa destructor* switching hosts from *A. cerana* to *A. mellifera* [23,24,25]. Whereas the mite is mostly harmless to *A. cerana*, it is lethal to *A. mellifera*, where it causes globally devastating colony losses in both managed and feral *A. mellifera* populations and is considered to be the largest threat to apiculture, wild, and feral honeybee populations [26].

More than three decades ago, the dwarf honeybee, *A. florea*, had been detected outside of its Asian endemic range. Lord and Nagi [27] reported on an *A. florea* population first detected in Khartoum (Sudan) in 1985. Until then, the *A. mellifera* was the only honeybee in Sudan with a large native wild population and a few managed honeybee populations kept in apiaries. Morphometric studies suggested that Pakistan was the country of origin of the introduced *A. florea* population in Sudan [28], and since the first sighting was near Khartoum International Airport, the incident was attributed to an accidental introduction via the airway [27,28].

In subsequent reports, it became clear that *A. florea* was not only transported by man, but naturally expanded from its endemic distribution range in Southeast Asia toward the West. Whereas occurrences in the warmer parts of Oman, Iran, and Pakistan were still outside the natural range of *A. mellifera* [13], the species is now also found in the Middle East, including Iraq and well-established sustainable populations on the Arabian Peninsula [29]. Most recently, *A. florea* has been reported in Eilat and Aqaba [30,31], and this population has spread recently to Egypt, marking a second entry into the African continent [32].

The introduction of *A. florea* into the range of *A. mellifera* is significant. *A. florea* is about 9 mm in body length, and about one-third the weight of a worker of *A. mellifera* [13]. The colonies are open nesting and construct only a single comb around a twig. They produce only 300–450 g of honey and the species is therefore only rarely used for honey production. The bees typically nest cryptic in bushes and are not very aggressive [14], and hence can stay undetected by man for a long time. Like many other tropical honeybees, *A. florea* is a migratory species that follows nectar flows with migratory swarms, and quickly absconds from its nest site if disturbed by predators or pests. When there is an ample food supply, the *A. florea* colony can send out multiple reproductive swarms [33]. It is, therefore, a highly mobile species with high reproductive potential, both of which are important prerequisites for any invasive species.

*A. florea* is the most widespread honeybee in most of tropical Asia [14]. *A. florea* is known to compete well with *A. mellifera* during foraging [34] and might even be robbing *A. mellifera* colonies [35,36]. Most importantly, however, are the potential diseases and pests carried by imported honeybees. *A. florea* honeybees are associated with the parasitic mite *Euvarroa sinhai* [37,38]. If these mites spill over to *A. mellifera* colonies, the results are unpredictable and may be as disastrous as in the case of *V. destructor*. Diseases are known to greatly facilitate invasive replacements, particularly if they are harmless to the invader but lethal to the resident species [39].

In this study, we assessed the invasive potential of *A. florea* in Sudan by following its spread northward along the river Nile. Because the Nile passes through desert regions, any survival of honeybees is bound to the river, and we could linearly study the spread with the river providing a natural transect. This allowed for clear predictions concerning the population’s genetic structure of the invading *A. florea*. Furthermore, we could assess any competition with native *A. mellifera* populations. If *A. florea* is detrimental for native honeybees, we would expect a negative correlation between the densities of native wild *A. mellifera* colonies and the imported *A. florea*. If *A. florea* has no major effect on *A. mellifera* densities, we would expect a positive correlation between both species. Because honeybee colonies of both species are extremely cryptic and hard to quantitatively detect in the field, we took advantage of the specific mating behavior of honeybees with drone congregation areas (DCA) and highly polyandrous queens. We can determine the number of drone-producing colonies in the local population via genotyping of the drones, either caught on a DCA or inferred from the queens’ worker offspring [40].

## 2. Materials and Methods

### 2.1. A. florea Worker Samples

**Figure 1:**
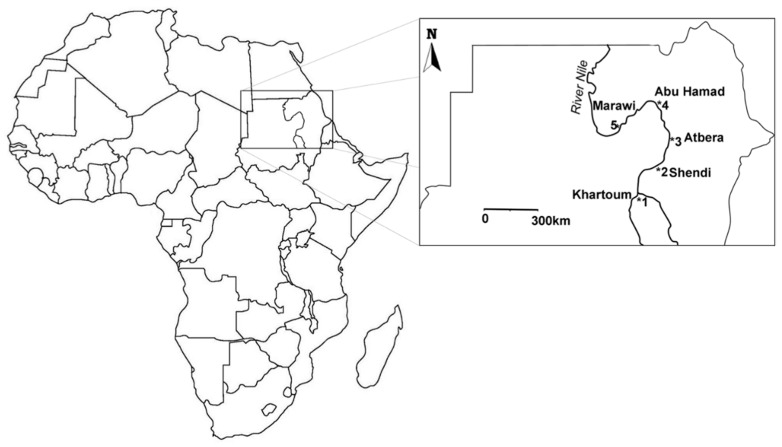
Map with the sampling sites of both *A. florea* and *A. mellifera* along the river Nile. Since we found no *A. mellifera* colonies at location 3, we collected drones at a local drone congregation area with a William’s trap. We found no *A. mellifera* bees at location 4, neither colonies nor drones.

Adult workers were collected from four *A. florea* colonies each at five locations starting from Khartoum (1) northward along the river Nile via Shendi (2), Adbera (3), Abu Hamad (4), and up to Marawi (5), 753 km away from Khartoum (Figure 1, coordinates in Table 1). Twenty-four workers were taken from each colony for DNA analyses. DNA was extracted from the hind leg using the Chelex^®^ (BioRad, Munich, Germany) method [41] and amplified with polymerase chain reactions (PCRs) using the protocol of Kraus et al. [42] with three already known microsatellite DNA loci—A76, A88, A107 [43,44]—and two additional loci—BI47 and AP19—both of which were used for the first time in *A. florea*. The queen and siring drone genotypes were determined from the worker genotypes using Mendelian inference as described by Moritz et al. [40].

### 2.2. A. mellifera Samples

We collected samples of *A. mellifera* from the same locations as the *A. florea* workers (Figure 1). Whenever we had access to colonies, we sampled 24 workers per colony. In Adbera, we found no *A. mellifera* colonies but we could collect drones at a local DCA using the William’s trap [45] with pheromone lures made of blackened cigarette filters and treated with about 10 queen equivalents of 9-oxodecenoic acid (2.5 mg) dissolved in dichloromethane. All the caught drones were immediately transferred into 95% EtOH until further processing for DNA extraction. DNA was extracted from all drones or 24 workers/colony using routine methods and genotyped with 5 tightly linked microsatellite loci on chromosome 13 (HB5, HB7, HB10, HB15, SV240) [46]. The use of closely linked loci greatly reduces the non-detection error (the probability of not identifying a mother queen due to two genotypes are identical by chance), because not only the occurrence of a given allele but the complete allele combination at all tested loci must be identical. Each queen produces only two drone genotypes with little recombination allowing for easy identification of the drones’ mothers [40,47].

### 2.3. Estimation of Population Density

Population densities were calculated based on the number of colonies detected and the mating flight range as in Moritz et al. [40] for *A. mellifera*. We estimated the population densities of *A. florea* in the same way since drone mating flight durations and queen mating flight times are similar in both species [48,49,50].

### 2.4. Genetic Structure of A. florea and A. mellifera Populations

After inferring the genotypes of the father drones, we used three parameters to calculate the mating frequency:

(1) the number of observed matings, *k_o_*, which underestimates the actual number of matings due to finite sample sizes,
(2) the estimated physical number of matings, *k_e_*, as given in Cornuet and Aries [51], to correct for differences in sample sizes, and
(3) the number of effective males, *m_e_* [52], which is based on the intracolonial relatedness among workers.

The expected heterozygosity, *H_E_* [53], and allelic richness, AR, were calculated from the drone allele frequencies for each subpopulation using FSTAT [54]. We calculated overall F_ST_-values using the allele frequency-based method of Weir and Cockerham [55] and a Fisher’s exact test for population differentiation [56]; both were implemented in Genepop [57].

## 3. Results

### 3.1. Polyandry

The five microsatellite loci (A76, A88, A107, BI47, AP19) used for genotyping the *A. florea* samples and the linked loci set for *A. mellifera* showed sufficient variability to conduct reliable polyandry analyses for determining the observed (*k_o_*), estimated (*k_e_*), and effective (*m_e_*) number of matings with low non-detection errors (smaller than 1%). For both species, the polyandry estimates were highly variable among colonies (Table 1). The average estimated number of matings was 7.9 ± 0.9 for *A. florea* (ranging from *k_e_* = 5 to *k_e_* = 15, *k_o_*: 5–12, *m_e_*: 3–19) and *k_e_* = 14.8 ± 1.8 for *A. mellifera* queens (ranging from *k_e_* = 9 to *k_e_* = 20, *k_o_*: 8–14, *m_e_*: 6–25). The average number of matings was significantly lower for *A. florea* queens (*k_e_* = 7.9 ± 0.9, *k_o_* = 7.1 ± 1.0, *m_e_* = 6.2 ± 1.1) than for *A. mellifera* queens (*k_e_* = 14.8 ± 1.7, *k_o_* = 11.5 ± 0.5, *m_e_* = 13.5 ± 0.2) for all values of the observed, estimated, and effective number of matings (*t*-test, *p* < 0.05).

### 3.2. Colony Density 

**Figure 2:**
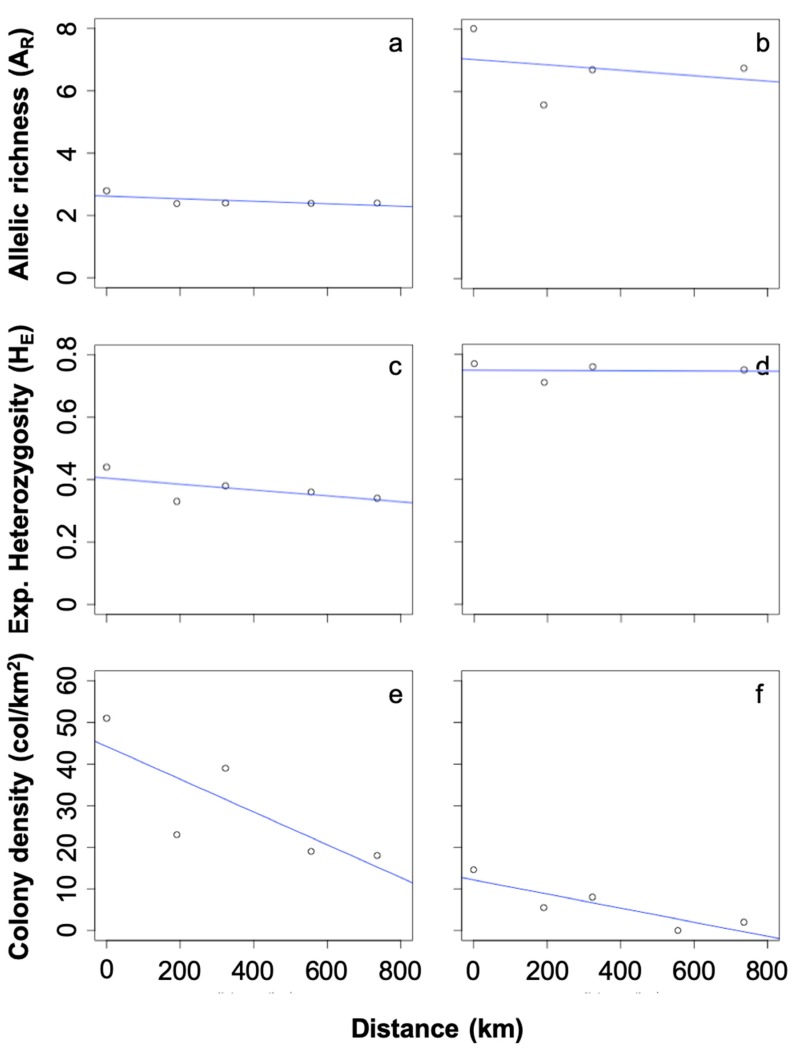
The decline of allelic richness (**a**,**b**), expected heterozygosity (**c**,**d**), and population density (**e**,**f**) from Khartoum (0 km) to Marawi (735.81 km) of both *A. florea* (left; a,c,e) and *A. mellifera* (right; b,d,f) along the river Nile.

**Figure 3:**
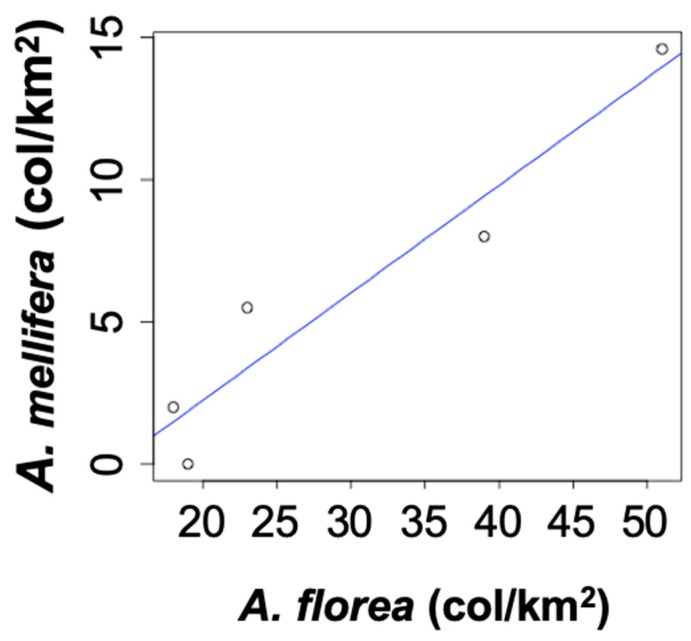
Correlation of colony densities between *A. florea* and *A. mellifera* along the river Nile valley sampling transect.

The density of *A. florea* colonies in the five sample locations ranged from 18 colonies/km² in Marawi to 51 colonies/km² in Khartoum (Table 1). In contrast, the colony density of *A. mellifera* ranged from 2 colonies/km² to 14.6 colonies/km² (Table 1) and was significantly smaller than the non-native *A. florea* populations (paired *t*-test, *p* < 0.03). Moreover, the densities of both *A. mellifera* and *A. florea* colonies showed significant decline northward along the transect (r^2^ = 0.75 and r^2^ = 0.63, respectively) (Figure 2) and a strong and highly significant positive correlation (r^2^ = 0.92, *p* < 0.01, Figure 3).

### 3.3. Population Genetic Structure 

The most important population genetic parameters characterizing both species are shown in Table 1. There were no cases in which we found significant deviations from the expected Hardy–Weinberg frequencies in both populations. The average expected heterozygosity was significantly smaller in the *A. florea* populations (HE = 0.37 ± 0.02) than in the *A. mellifera* populations (HE = 0.74 ± 0.02; *t*-test, *p* = 0.019). Similarly, the mean frequencies of heterozygotes estimated from the derived queen genotypes were significantly smaller in *A. florea* (HE = 0.31 ± 0.03) than in *A. mellifera* (HE = 0.76 ± 0.04) populations. The average allelic richness in the *A. florea* populations (AR = 2.47 ± 0.09) was significantly lower than in the *A. mellifera* populations. (AR = 6.8 ± 1.001; *t*-test, *p* < 0.001, Table 1). The allelic richness of both *A. mellifera* and *A. florea* populations significantly declined on the northward transect along the river Nile going northward (r^2^ = 0.79 and r^2^ = 0.55, respectively, Figure 2).

Despite a low overall FST = 0.033 among all subpopulations of *A. florea*, a Fisher’s exact test showed a highly significant overall genetic differentiation among the sample locations. However, in pairwise comparisons between all populations (e.g., sample location), only 4 out of 10 pairs showed a highly significant differentiation (a combination of Shendi with other locations).

### 3.4. Estimation of the Number of A. florea Colonies Introduced to Khartoum

To estimate the minimal number *A. florea* colonies initially introduced to Khartoum, we tested whether the total number of alleles found in the entire *A. florea* sample along the river Nile could have originated from a single colony. Since queens of *A. florea* mate on average with eight drones, a single colony should contain a maximum number of 10 alleles (2 queen alleles + 8 males’ alleles) assuming complete independence of the males. We found an average of only 2.7 ± 0.2 alleles per locus in the entire *A. florea* population in Sudan, which can easily be present in a single colony. However, this was an extremely conservative approach and it might be more meaningful to take the actual genetic variability in endemic *A. florea* populations into account. The number of alleles is finite and it is unlikely that each drone carries a different allele at all the tested loci. Using the data of Palmer and Oldroyd [44] for three loci of native *A. florea* in Thailand, we estimated an average of 2.20 ± 0.51 alleles per locus in a single *A. florea* colony (Table 2). This comprises a more realistic value of the number of alleles per locus per colony. However, even considering this conservative estimate, the total number of alleles per locus for the *A. florea* colonies in the entire sample along the river Nile was only slightly higher and not significantly different.

## 4. Discussion

Although, *A. florea* can have similar mating frequencies to *A. mellifera* [44,58], we found that the average degree of polyandry of *A. florea* queens was significantly less than that of *A. mellifera* queens in Sudan. This was not related to a lack of drones due to too few colonies because we found a much higher colony density in *A. florea* populations than in *A. mellifera* populations at all sampling locations along the river Nile. We also failed to find indications of interspecies competition. Any strong competition between *A. florea* and *A. mellifera* should have caused a negative correlation in population densities between both species. However, we found a positive correlation between the population densities of the two species. Certainly, the northward spread of *A. florea* did not cause a detectable decline in the population density of the native *A. mellifera*. The population densities of both species markedly declined in the more northern sampling locations, suggesting that factors other than interspecies competition contributed to this decline. As the vegetation degraded from a dry savannah near Khartoum to desert in the North with only very light and irregular rainfall (0–50 mm per year), it is only directly along the river Nile where honeybees can survive. The further north one goes, the narrower the strip of suitable habitats, and the reduction in habitat size may be the main driver of the northward decline of the honeybee population in Sudan. Although there have been reports of competitive foraging between *A. florea* and other *Apis* species in Asia [38], this was not observed in Sudan [59]. All *A. florea* samples were free of known parasitic mites and other typical pests and diseases of honeybees [60]; hence, there was no evidence that pathogen spill overs might have interfered with species competition.

Introduced bees have been claimed to alter the population structure of plants by mediating pollination and increasing the seed set of invasive weeds [39]. However, to our knowledge, there have been no reports that *A. florea* has had any negative impact on biodiversity, ecosystem, agriculture, or the public in Sudan. In contrast, the honey of *A. florea* has been adopted for use in traditional medicine and it is considered superior in quality. Furthermore, *A. florea* is an efficient pollinator, especially of cotton [59].

### Population Genetic Structure

Our data show that the non-native *A. florea* had higher population densities despite a reduced genetic diversity compared to the native *A. mellifera*. This may reflect the ability of the *A. florea* to expand and reproduce more rapidly than the cave-breeding Western Honeybee. Akratanakul [33] reported that *A. florea* colonies send out multiple reproductive swarms when there is ample food supply. Furthermore, *A. florea* appeared to be free of parasites in Sudan [60] and hence could spread free of parasitization, predation, and competition in the new habitat [59]. In particular, the lack of pests and disease might have facilitated the swift spread of *A. florea*. The highly flexible nesting behavior of *A. florea* colonies, which are readily found in human houses and gardens, might be another feature supporting the high population densities. Since colonies are not very aggressive, they often remain undetected.

Sudan is a diversity hot spot for *A. mellifera*, comprising three different evolutionary lineages (A, O, and C) with four recognized native *A. mellifera* subspecies (*A. m. lamarckii*, *A. m. syriaca*, *A. m. scutellata*, *A. m. jemenitica* [61]). Hence, it is not surprising that we found a high genetic diversity in the sampled *A. mellifera* populations. The reduced genetic diversity and allelic richness of *A. florea* was probably due to the very small introduced *A. florea* population. Our data suggest that the origin of introduction was in or south of Khartoum. The highest number of alleles was found in Khartoum, suggesting that alleles were lost by genetic drift on the northward spread.

The entire *A. florea* population north of Khartoum comprised of an average of 2.7 alleles per locus. This is very similar to the number of alleles found in a single colony in endemic *A. florea* colonies in Asia [44]. This value also compares well with the genetic variability in the *A. florea* population that has recently spread in Israel and Jordan, which has also been attributed to a single colony introduction [31]. In this regard, we cannot exclude the possibility that the *A. florea* population in Sudan originated from the introduction of a single colony more than three decades ago.

This raises a question regarding how the introduced bees deal with the genetic load at the sex locus, i.e., the gene responsible for the sex determination, which gives rise to females when heterozygous and to males when hemi- or homozygous [62]. Homozygous males are non-viable and are parasitized by their sisters. This high genetic load results in negative frequency-dependent selection resulting in a hyper-allelic locus. During similar invasion events by the Eastern honeybee *A. cerana*, which established itself from a single introduced colony in Australia [63], the colony profited from a system of multiple mating [64], which allows a colony to maintain a high number of different sex alleles. Thus, invasions of single colonies can lead to the establishment of stable populations supported by the multiple mating of honeybees, which provides sufficient genetic material for natural selection to act on, thereby reducing the detrimental effects of population bottlenecks.

## 5. Conclusions

The dwarf honeybee *Apis florea* is has been detected in 1985 in Khartoum, Sudan, for the first time on the African continent and has spread along the river Nile. It is coexisting with the native *A. mellifera*. The original introduction traces back to a single colony, but due to multiple mating sufficient genetic material is present to overcome the genetic load at the sex locus.

## Figures and Tables

**Table 1:** Genetic structure of the sampled *A. florea* and *A. mellifera* populations. N = number of colonies. *k_e_* = estimated number of matings, AR = allelic richness, col/km² = colony density, *H_E_* = expected heterozygosity, n = number drones inferred from worker sample, * physical drone samples on DCA (ke not applicable).

***A. florea.***									
**Location**	**N**	**Distance to Khartoum (km)**	***k_e_***	**AR**	**col/km^2^**	***H_E_***	**n**	**North**	**East**
Khartoum	4	0	11.5 ± 1.5	2.79	51.0	0.44	32	15°35′	32°32′
Shendi	4	191.2	8.7 ± 1.3	2.38	23.0	0.33	25	16°42′	33°26′
Adbera	4	323.2	7.0 ± 2.4	2.40	39.0	0.38	25	17°41′	33°58′
Abu Hamad	4	555.9	6.2 ± 1.1	2.39	19.0	0.36	16	19°31′	33°19′
Marawi	4	753.8	6.2 ± 0.1	2.40	18.0	0.34	20	18°28′	31°49′
Total	20						118		
Mean ± SE			7.9 ± 0.9	2.47 ± 0.09	30.0 ± 7.2	0.37 ± 0.02			
***A. mellifera***									
Khartoum	4	0	16.5 ± 1.4	8.02	14.6	0.79	58	15°35′	32°32′
Shendi	4	191.2	13.0 ± 1.2	5.57	5.5	0.71	52	16°42′	33°26′
Adbera*	n.a.	323.2	n.a.	6.70	8.0	0.76	72	17°41′	33°58′
Abu Hamad	n.a.	555.9	n.a.	n.a.	n.a.	n.a.	n.a.	19°31′	33°19′
Marawi	2	753.8	14.7 ± 1.7	6.75	2.0	0.75	60	18°28′	31°49′
Total	10						182		
Mean ± SE			14.8 ± 1.8	6.8 ± 1.001	7.5 ± 5.3	0.75 ± 0.03			

**Table 2:** Number of alleles in native and introduced colonies of *A. florea*. The average number of alleles found in the entire population of *A. florea* in Sudan did not significantly exceed that found in a single colony of *A. florea* from its original region (data from Thailand obtained from Palmer and Oldroyd [44]).

Thailand Population						
Locus Name	Number of Alleles in Each Colony					
	1	2	3	4	5	Mean
A76	2	1	1	1	1	1.20
A88	4	2	3	2	2	2.60
A107	3	3	2	3	3	2.80
Mean ± SE	3	2	2	2	2	2.20 ± 0.50
**Khartoum Population**						
A76	3	2	2	3		2.50
A88	2	3	1	2		2.00
A107	3	3	3	2		2.75
Mean ± SE	2.66	2.66	2	2.33		2.42 ± 0.22
**Sudan:Number of Alleles in Each Location**						
	Khartoum	Shendi	Adbera	Abu-Hamad	Marawi	Mean
A76	3	3	3	3	3	3.00
A88	3	2	2	2	2	2.20
A107	3	3	3	3	3	3.00
Mean ± SE	3	2.66	2.66	2.66	2.66	2.73 ± 0.22
